# The quality of economic evaluations of ultra-orphan drugs in Europe – a systematic review

**DOI:** 10.1186/s13023-015-0305-y

**Published:** 2015-07-30

**Authors:** Y. Schuller, C. E. M. Hollak, M. Biegstraaten

**Affiliations:** Department of Endocrinology and Metabolism, Academic Medical Center, University of Amsterdam, F5-166, P.O. Box 22660, , 1100 DD Amsterdam, The Netherlands

**Keywords:** Rare diseases, Orphan drugs, Cost-effectiveness, Economic evaluation, Costs and cost analysis

## Abstract

An orphan disease is defined in the EU as a disorder affecting less than 1 in 2 000 individuals. The concept of ultra-orphan has been proposed for diseases with a prevalence of less than 1:50 000. Drugs for ultra-orphan diseases are amongst the most expensive medicines on a cost-per-patient basis. The extremely high prices have prompted initiatives to evaluate cost-effectiveness and cost-utility in EU-member states. The objective of this review was to evaluate the quality of cost-effectiveness and cost-utility studies on ultra-orphan drugs. We searched 2 databases and the reference lists of relevant systematic reviews. Studies reporting on full economic evaluations, or at least aiming at such evaluation, were eligible for inclusion. Quality was assessed with the use of the Consensus on Health Economic Criteria (CHEC)-list. Two-hundred-fifty-one studies were identified. Of these, 16 fitted our inclusion criteria. A study on enzyme replacement and substrate reduction therapies for lysosomal storage disorders did not perform a full economic evaluation due to the high drug costs and the lack of a measurable effect on either clinical or health-related quality of life outcomes. Likewise, a cost-effectiveness analysis of laronidase for mucopolysaccharidosis type 1 was considered unfeasible due to lack of clinical effectiveness data, while in the same study a crude model was used to estimate cost-utility of enzyme replacement therapy (ERT) for Fabry disease. Three additional studies, one on ERT for Fabry disease, one on ERT for Gaucher disease and one on eculizumab for paroxysmal nocturnal haemoglobinuria, used an approach that was too simplistic to lead to a realistic estimate of the incremental cost-effectiveness (ICER) or cost-utility ratio (ICUR). In all other studies (*N* = 11) more sophisticated pharmacoeconomic models were used to estimate cost-effectiveness and cost-utility of the specific drug, mostly ERT or drugs indicated for pulmonary arterial hypertension (PAH). Seven studies used a Markov-state-transition model. Other models used were patient-level simulation models (*N* = 3) and decision trees (*N* = 1). Only 4 studies adopted a societal perspective. All but 2 studies discounted costs and effects appropriately. Drugs for metabolic diseases appeared to be significantly less cost-effective than drugs indicated for PAH, with ICERs ranging from €43 532 (Gaucher disease) to €3 282 252 (Fabry disease). Quality of studies using a Markov-state-transition or patient-level simulation model is in general good with 14–19 points on the CHEC-list. We therefore conclude that economic evaluations of ultra-orphan drugs are feasible if pharmacoeconomic modelling is used. Considering the need for modelling of several disease states and the small patient groups, a Markov-state-transition model seems to be most suitable type of model. However, it should be realised that ultra-orphan drugs will usually not meet the conventional criteria for cost-effectiveness. Nevertheless, ultra-orphan drugs are often reimbursed. Further discussion on the use of economic evaluations and their consequences in case of ultra-orphan drugs is therefore warranted.

**Electronic supplementary material:**

The online version of this article (doi:10.1186/s13023-015-0305-y) contains supplementary material, which is available to authorized users.

## Introduction

In the European Union (EU), a disease is considered ‘orphan’ if it is a life-threatening or seriously debilitating disorder that affects fewer than 1 per 2 000 (or less than 0.05 %) of the population [1]. To date, 7 000 rare diseases have been identified, affecting 30–40 million patients in the EU [2]. Although no legal definition of ‘ultra-orphan’ diseases has been established, this subcategory was introduced by the National Institute for Health and Care Excellence (NICE). It is suggested to be applied to diseases with an estimated prevalence of <1:50 000 [3]. Initially, the small consumers’ market of orphan drugs restrained the pharmaceutical industry from developing medicines for rare diseases. For this reason, European legislation was introduced in 2000 to stimulate the development of orphan drugs, following the example of the United States who introduced the Orphan Drug Act in 1983. This legislation implies that the pharmaceutical industry has a right to i) obtain protocol assistance at a reduced charge, ii) gain access to the centralized authorization procedure, iii) get reduction of registration costs, and iv) benefit from 10 years of market exclusivity after registration [4]. This has led to the authorization of 124 new orphan drugs in the EU from 2000 until 2015 by the European Medicines Agency (EMA), of which about one-third is indicated for an ultra-orphan disease (http://www.ema.europa.eu/ema/). Authorization of these ultra-orphan drugs is often based on studies in small patient groups not representing the entire patient population. Moreover, biomarkers and intermediate endpoints are often used [5]. Because of this, longer term effectiveness on clinically relevant outcome measures is often unclear at the time of authorization. If, at time of authorization, insufficient evidence on efficacy and safety exists, the regulatory authorities can register an orphan drug by approving it ‘under exceptional circumstances’. This applies to drugs for which the applicant can demonstrate that comprehensive data cannot be provided (due to specific reasons foreseen in the legislation). At the same time, costs of orphan drugs have increased over the past years, in particular costs of ultra-orphan drugs. This rise has to do with the rapid technical advances including unravelling of molecular mechanisms that underlie some diseases and require a more personalised approach [6]. About 250 new rare diseases are described annually [7]. For member states to make decisions on reimbursement, it is important to gain a better insight into the balance between expenses and health gains for a specific drug in order to determine the ‘value for money’ for orphan drugs. Consequently, economic evaluations play an important role in current decision making about the reimbursement of drugs. In some countries in the EU, reimbursement of drugs depends on the incremental costs per quality-adjusted life year (QALY). In England and Wales, incremental costs exceeding £20 000 to £30 000 per QALY gained are in principle not reimbursed [8–10]. Conversely, other countries in the EU recommend against any pre-set threshold. It is, however, a scientific challenge to evaluate the cost-effectiveness or cost-utility of orphan drugs. With this review we aim to assess the economic evaluations ultra-orphan drugs marketed in the EU that have been performed so far with specific focus on the methods used and the quality of the studies.

## Methods

The EMA created the ‘List of medicinal products for rare diseases in Europe’ (http://ec.europa.eu/health/documents/community-register/html/index_en.htm), which describes orphan medicinal products (OMPs) in Europe with European orphan designation and marketing authorization. We used this list, which was updated in April 2015, to identify all OMPs in Europe. Subsequently, www.orpha.net was searched to select those drugs that were developed to treat ultra-orphan diseases (prevalence < 1:50 000).

For each OMP included in this study, we conducted a literature search in the databases of MEDLINE and EMBASE (both via OvidSP) for economic evaluations published prior to April 2015. Key terms used included the disease indication, the drug’s generic name and EU brand name, and the terms cost-benefit, cost-effectiveness, health economics, pharmaco-economics, economic evaluation and quality-adjusted life years, including alternative notations. Additional reports were identified by hand searching the reference lists in the retrieved papers. For each literature search, one author (YS) read the titles and abstracts of all identified studies. If considered relevant, full text was read and analysed. Papers that met the following inclusion criteria were included: I) reporting on an original cost-effectiveness or cost-utility analysis, or at least aiming at such analysis, of an orphan drug for its approved orphan indication, II) orphan drug is marketed in the EU and is indicated for a condition with a prevalence of <1:50 000 (ultra-orphan drug), and III) article is published in the English language. OMPs withdrawn from the EU market or discontinued from the community register of orphan medicinal products (at the end of the 10-year period of market exclusivity) were also included. Studies in which data on costs and/or utilization were collected but not related to a measure of benefit were excluded. To convert different currencies used in the included studies, we used a currency converter (May 2015).

**Fig. 1:**
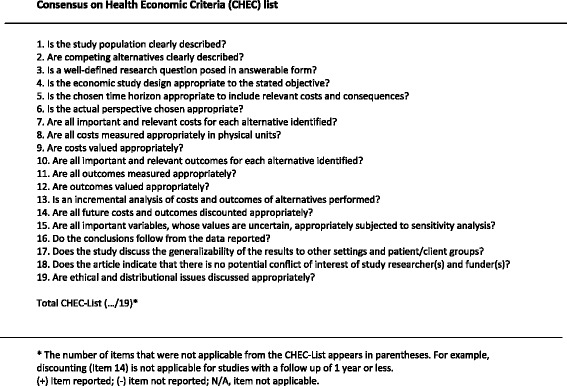
Consensus on Health Economic Criteria (CHEC) list

We used the 19-item Consensus on Health Economic Criteria (CHEC)-list to assess the quality of economic evaluations (see Fig. 1) [11]. The CHEC-list has been developed using a Delphi method and focuses on the methodological quality of economic evaluation aspects. It was developed for systematic reviews of full economic evaluations based on effectiveness studies (cohort studies, case–control studies, randomized controlled trials) and is recommended by the Cochrane Handbook for Systematic Reviews of Interventions [12]. The CHEC rating and data extraction was conducted by two authors (YS and MB).

## Results

### Literature search

**Fig. 2:**
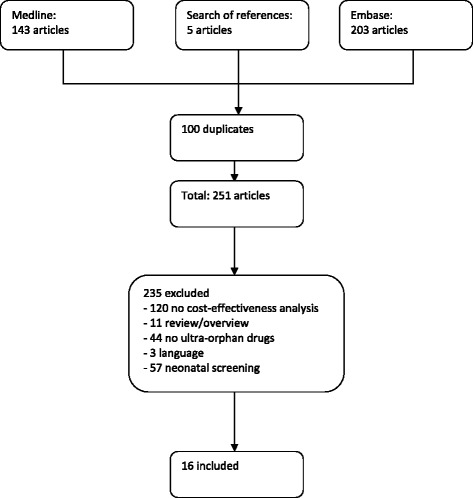
Study flow diagram

We identified 32 ultra-orphan drugs from the EMA and Orphanet website. The cost-utility study for type 1 Gaucher disease compares ERT (imiglucerase [Cerezyme®] and velaglucerase alfa [Vpriv®]) with standard medical care [13]. Although imiglucerase is not registered as an (ultra) orphan drug due to the fact that the EU orphan legislation was installed at a later stage, we decided to include this study in our review since Gaucher is considered to be an ultra-orphan disease. The same applies to the study by Beutler et al., which compares alglucerase (Ceredase®) for Gaucher disease to standard of care [14]. The literature search resulted in 251 articles, of which 16 met our inclusion criteria (see Fig. 2). The majority of the studies considered treatments of lysosomal storage disorders (LSDs) and pulmonary arterial hypertension (PAH). Two of the 16 articles reported on more than 1 ultra-orphan drug resulting in 19 economic evaluations in total (see Table 1). The table summarizes the methods used, comparators, base-case incremental cost-effectiveness ratios (ICERs)/cost-utility ratios (ICURs), and CHEC-list sum scores. To estimate the ICER/ICUR of ultra-orphan drugs marketed for LSDs (alglucosidase alfa [Myozyme®], agalsidase alfa [Replagal®], algasidase beta [Fabrazyme®], alglucerase, imiglucerase, velaglucerase alfa and mercaptamine [Procysbi®]) and for paroxysmal nocturnal haemoglobinuria (eculizumab [Soliris®]), these drugs have been compared to standard medical care, while ultra-orphan drugs for PAH (sildenafil [Revatio®], iloprost [Ventavis®], bosentan [Tracleer®] and ambrisentan [Volibris®]) have been compared with each other. Agalsidase alfa, agalsidase beta, laronidase, sildenafil, iloprost and bosentan were approved under exceptional circumstances. The majority of the studies included were conducted in the USA [14–18], followed by the UK [19–23], and the Netherlands [13, 24, 25]. The remaining studies have been conducted in Spain [26], Australia [27] and Canada 28].

**Table 1:** Results of included studies

Drug	Indication	Prevalence	Comparator(s)	Economic model	Base-case results per QALY, discounted	Study characteristics and limitations	CHEC score	Reference
ERT/SRT	LSDs	<1: 50 000	soc	No cost-effectiveness analyses were undertaken owing to the high drug costs and the lack of measurable effect on either clinical or health-related quality of life outcomes. Combined with the current annual price of the different ERTs, between 3.6 and 17.9 discounted QALYs would need to be generated for each year of being on treatment in order for them to be considered cost-effective (or between 2.6 and 10.5 discounted QALYs for children).				Wyatt [22]
Laronidase (Aldurazyme)	MPS1	1: 100 000	soc	No cost-effectiveness model was developed due to lack of data.				Connock [21]
Agalsidase alfa and beta (Fabrazyme and Replagal)	Fabry disease	0.22: 100 000	soc	Markov-state-transition model	€3 282 252	Eleven Markov states were defined, including no symptoms, one or multiple complications and death, with a 1-year cycle period and a time horizon of 70 years. TPs, utilities and costs (both direct and indirect) were derived from retrospective and prospective follow-up data of the Dutch cohort. Utilities and costs were assumed to be equal in both arms as long as patients are in the same disease state, except for ERT costs.	18	Rombach [25]
Agalsidase alfa and beta (Fabrazyme and Replagal)	Fabry disease	0.22: 100 000	soc	Not specified	€351 622^b^	Limited information reported in the literature precluded the development of a comprehensive model. The decision model considered a birth cohort of male patients. Survival and utilities for untreated patients were estimated from literature. ERT was assumed to restore patients to full health and no disease-specific mortality. Only direct costs were considered.	13	Connock [21]
Agalsidase alfa and beta (Fabrazyme and Replagal)	Fabry disease	0.22: 100 000	soc	Not specified	80 % probability that ERT has a positive net benefit at a willingness to pay per QALY of €266 520^b^	Utilities were estimated on the basis of 2 studies. Costs were roughly estimated without using references. Bootstrapping was used to obtain ICER distributions. Results were plotted in a cost-effectiveness acceptability-curve. Primary objective of the study was not to calculate the ICER but to draw upper and lower limits of the current pricing structure.	8	Moore [16]
Velaglucerase alfa and imiglucerase (Vpriv and Cerezyme)	Gaucher disease	1: 100 000	soc	Markov-state-transition model	€432 540	Eight Markov states were defined, including no symptoms, one or multiple complications and death, with a 1-year cycle period and a time horizon of 85 years. TPs, utilities and costs (both direct and indirect) were derived from retrospective and prospective follow-up data of the Dutch cohort. Utilities and costs were assumed to be equal in both arms as long as patients are in the same disease state, except for ERT costs.	19	Van Dussen [13]
Alglucerase (Ceredase)	Gaucher disease	1: 100 000	soc	Not specified	€43 532^b^ (2.3U/kg three times weekly)	Estimate of ICERs for 3 drug dosing regimens was based on the assumption that alglucerase results in survival with perfect quality of life vs immediate death without treatment.	7	Beutler [14]
					€66 631^b^ (30U/kg/2wk)			
					€130 595^b^ (60U/kg/2wk)			
Alglucosidase alfa (Myozyme)	Pompe disease (infantile)	1: 14 000 to	soc	Patient-level simulation model	€1 043 868	Survival in both treatment arms was estimated on the basis of international studies, literature and their own patient cohort. Utilities and costs were derived from 6 and 12 patients resp., and were assumed to be equal in both arms except for ERT and infusion-related costs.	17	Kanters [24]
		1: 300 000						
Alglucosidase alfa (Myozyme)	Pompe disease (infantile)	1: 14 000 to	soc	Markov-state- transition model	England: €326 791^b^	Two Markov states were defined: alive-symptomatic and dead, with a 1-year cycle and a time horizon of 20 years. Survival rate (75 vs 8 %) and costs were derived from literature and historic databases. It was assumed that ERT would result in higher utilities. Only direct costs were considered. Costs and QALYs were generated for 2 settings: England and Colombia.	16	Castro-Jaramillo [19]
		1: 300 000			Colombia: €153 405^b^			
Eculizumab (Soliris)	Paroxysmal Nocturnal Haemoglobinuria	1: 500 000	soc	Markov-state-transition model	€1 620 256^b^	Six different consequences from PNH or treatment were modelled. Patients may have more than 1 complication at the same time, resulting in a total of 47 disease states. Analysis was conducted for 6 patient strata to account for differences in thrombosis risk and transfusion requirements. TPs for both arms were estimated on the basis of cohort studies and clinical trials. Utilities were derived from literature. Only direct costs were considered.	17	Coyle [28]
Eculizumab (Soliris)	Paroxysmal Nocturnal Haemoglobinuria	1: 500 000	soc	Not specified	1) €358 655 (haemoglobin) €474 998 (LDH)	Due to the lack of reliable quantitative information about costs and benefit of eculilzumab, a fully informed estimation of cost-effectiveness was not feasible. Instead, 3 preliminary analyses were conducted to estimate the costs per: 1) patient with stabilised of haemoglobin and normalised LDH, 2) LYG under the assumption that eculizumab returns survival to normal, and 3) LYG based on reduced mortality rates from thrombosis.	9	Connock [28]
					2) €697 500 - €1 953 000/LYG			
					3) €3 906 000 - €4 464 000/LYG			
Mercaptamine (Procysbi)	Cystinosis	<1: 50 000	soc	Decision tree	Dominant; cysteamine is more effective and less expensive	Drug studied is cysteamine (Cystagon), which is the ‘reference drug’ for mercaptamine, i.e., both drugs are equal. Time to first renal failure was estimated to be 10 years for untreated and 15 years for treated patients based on literature. After first renal failure all patients were assumed to follow the same disease course. Only direct costs were considered. Costs were discounted, outcomes were not. Hence life expectancy increases by 5 years while costs for dialysis and transplants are delayed resulting in cost savings.	15	Soohoo [17]
Sildenafil (Revatio)	PAH	5: 1 000 000	one of: bosentan, treprostinil, epoprostenol, ilioprost, sitaxentan	Markov-state-transition model	Dominant; sildenafil is more effective and less expensive	Five states were defined (WHO functional classes and death), with a 3-month cycle period and a time horizon of 1 year. The model was based on 100 hypothetical patients with a gender and disease severity distribution based on previous studies. TPs were based on 3-months transitions reported for bosentan and estimated for the other drugs after adjustment for improvement in 6MWT. Reported utility values for each functional class were used, adjusted for route of administration. Only direct costs were considered.	15 (2)^a^	Garin [15]
Iloprost (Ventavis)	PAH	5: 1 000 000	treprostinil	Markov-state-transition model	€1 228 055^b^	See description above for Garin [15]	15 (2)^a^	Garin [15]
			epoprostenol		€1 016 649^b^			
Iloprost (Ventavis)	PAH	5: 1 000 000	treprostinil	Markov-state-transition model	Dominant; iloprost is more effective and less expensive	Five Markov states were defined representing the WHO functional classes and death, with a 3-month cycle period and a time horizon of 3 year. Treatment changes were allowed if patient status worsened. The model was based on 100 hypothetical patients with functional class III. TPs were based on 6MWT results obtained from clinical trials, utilities from the literature. Only direct costs were considered. All parameters were validated by an expert opinion.	15 (1)^a^	Roman [26]
			epoprostenol vs iloprost		€6 847 284			
Bosentan (Tracleer)	PAH	5: 1 000 000	soc	Patient-level simulation model	Dominant; bosentan is more effective and less expensive	Time to progression functional class III to IV and utilities were estimated on the basis of international studies and literature. Life expectancy was assumed to be independent of the initial treatment. Time spent in the most severe disease state (with higher costs due to continuous treatment with prostaglandins in this state, and lower utility) is thus the only varying outcome parameter between the 2 treatment arms. Only direct costs were considered. 10,000 patients were simulated and replicated. For each patient a discrete event simulation approach was used in which the simulated time moved directly to the time of the next event (progression or death).	14 (1)^a^	Stevenson [20]
Bosentan (Tracleer)	PAH	5: 1 000 000	one of: epoprostenol, treprostinil	Markov-state-transition model	Dominant; bosentan is more effective and less expensive	Five Markov states were defined representing the WHO functional classes and death, with a 3-month cycle period and a time horizon of 1 year. The model was based on 100 hypothetical patients. TPs were based on 3-months transitions reported for bosentan and estimated for the other drugs after adjustment for improvement in 6MWT. Utilities were estimated by a group of clinical experts. Only direct costs were considered.	14 (2)^a^	Highland [18]
Bosentan (Tracleer)	PAH	5: 1 000 000	soc	Patient-level simulation model	€40 137 / LYG^b^	Mortality rate in both treatment arms was estimated on the basis of international studies and literature. The key assumption was that bosentan increases life expectancy. Only direct costs were considered. 5,000 patients were simulated and replicated. At 6-months intervals the patient progressed through one or more health states (bosentan, conventional therapy or death) with a 15-year time horizon.	14 (1)^a^	Wlodarczyk [27]
Bosentan (Tracleer)	PAH	5: 1 000 000	one of: epoprostenol, iloprost	Markov-state-transition model	Dominant; bosentan is more effective and less expensive	See description above for Garin [15]	15 (2)^a^	Garin [15]
			treprostinil		€65 282^b^			
			sitaxentan		€2 621^b^			
Ambrisentan (Volibris)	PAH	5: 1 000 000	one of: iloprost, epoprostenol	Markov-state-transition model	Dominant; ambrisentan is more effective and less expensive	See description above for Garin [15]	15 (2)^a^	Garin [15]
			bosentan		Equal; no difference in effectiveness and costs			
			treprostinil		€65 282^b^			
			sildenafil		Dominated; ambrisentan is equally effective but more expensive			
			sitaxentan		€2 621^b^			

*ERT* enzyme replacement therapy, *SRT* substrate reduction therapy, *LSD* lysosomal storage disorder, *MPS* mucopolysaccharidosis, *PAH* pulmonary arterial hypertension, *ICER* incremental cost-effectiveness ratio, *QALY* quality adjusted life year, *LYG* life year gained, *soc* standard of care, *TP* transition probability, *6MWT* 6-minute walk test

^a^ The number of items that were not applicable from the CHEC-List appears in parentheses. For example, discounting is not applicable for studies with a follow up of 1 year or less

^b^ Currencies converted by http://www.convertmymoney.com/ on May 1^st^ 2015

### Economic models

A study on enzyme replacement and substrate reduction therapies for LSDs did not conduct a full economic evaluation since a cost-effectiveness or cost-utility analysis was considered unfeasible due to the high drug costs and the lack of a measurable effect on either clinical or health-related quality of life outcomes [22]. In spite of this, the authors calculated that 3.6–17.9 discounted QALYs would need to be generated for each year of being treated in order for them to be cost-effective. Likewise, a cost-effectiveness analysis of laronidase [Aldurazyme®] for mucopolysaccharidosis type 1 was not performed as a consequence of limited evidence of effectiveness [21]. In the same study, a crude decision model was used to estimate cost-utility of agalsidase alfa and beta for Fabry disease. A perfect drug scenario was assumed (i.e., treatment with agalsidase alfa or beta was assumed to restore patients to full health), which undoubtedly led to an underestimation of the ICUR (€351 622 per QALY). The same holds true for another study on enzyme replacement therapy (ERT) for Fabry disease [16]. This study roughly estimated health gains based on only 2 studies, of which 1 reported utilities of Fabry patients in the pre-ERT era and the other used data from the Fabry Outcome Survey (FOS, a Shire sponsored post-marketing drug registry) 10 years later. It is known, however, that the availability of ERT prompted awareness programs and screening studies which ultimately led to an expansion of the disease’s phenotype. Consequently, the FOS cohort consists of patients who have on average a milder disease course than the patients in the pre-ERT study, making it incorrect to ascribe the higher utility scores to the effect of ERT. The reported 80 % probability that ERT has a positive net benefit at a willingness-to-pay of €266 520 per QALY is therefore an overestimation. Cost-utility of alglucerase for Gaucher disease was roughly estimated without using a comprehensive economic model. The authors assumed the best effect a drug can have (immediate death versus survival with perfect quality of life) which certainly led to an underestimation of the ICUR (€43 532–€130 595 per QALY depending on the dose used) [14]. Finally, Connock et al. conducted three crude economic evaluations. Under the assumptions that eculizumab returns survival to normal, the ICER varied from €697 500–€1 953 000per life year gained [23]. In all 4 studies an imprecise estimate of costs was made [14, 16, 21, 23].

The remaining 11 studies used more sophisticated pharmacoeconomic models to evaluate costs and effects of a certain treatment, of which 7 used a Markov-state-transition model [13, 15, 18, 19, 25, 26, 28]. Other models used were patient-level simulation models (*N* = 3) [20, 24, 27] and decision trees (*N* = 1) [17]. In only 1 study the scientific basis of the model was explained [20]. In this study, a patient-level simulation model was used because the authors found it more computationally efficient and it removed the need for establishing arbitrary durations for the time cycles as required by a Markov model. In general, pharmacoeconomic models use data from different sources such as literature, own patient cohorts and clinical experience, and are therefore able to give more precise estimates than the studies mentioned above.

### Quality assessment

Quality scoring of studies that conducted a cost-effectiveness or cost-utility analysis varied from 7 to 19 out of 19 points on the CHEC-list, with studies using a very simple model having lower scores (7–13) [14, 16, 21, 23] than studies using a Markov-state-transition or patient-level simulation model (14–19) [13, 15, 18–20, 24–28]. The study that used a decision tree scored 15 points [17].

Data on costs and effects were mostly obtained from literature, hospital databases, and health and labour questionnaires. The studies that used a patient-level simulation model restricted events to clinical deterioration and/or death [20, 24, 27], while Markov-state-transition models used up to 47 disease states to describe the course of the disease under study [28]. All studies measured outcomes in QALYs (cost-utility analyses), except for 2 studies in which costs per life year gained was calculated [23, 27]. Four studies considered a societal perspective [13, 17, 24, 25], while the other studies considered only direct medical costs (i.e., a health care perspective) [14–16, 18–21, 23, 26–28]. In all but 2 [16, 17] studies costs and effects were discounted appropriately. The studies by Highland and Garin did not require discounting as the time horizon was 1 year [15, 18]. In most studies the ICER/ICUR was calculated. If a drug is both clinically superior and less expensive, the drug under study is referred to as a ‘dominant’ drug. The opposite is a ‘dominated’ drug, which applies to ambrisentan in the study by Garin et al. [15]. In both cases of dominancy, the ICER would have reached a negative value and is usually not calculated. Thirteen studies applied a sensitivity analysis [13, 15–21, 24–28]. Eight studies discussed the issue of generalizability for application in a different context [13, 15–19, 21, 27]. CHEC-list scores of individual studies are shown in Additional file 1.

### Incremental cost-effectiveness/cost-utility ratios

The ICERs and ICURs of individual ultra-orphan drugs are depicted in Table 1. In general, drugs for metabolic diseases appeared to be significantly less cost-effective than drugs indicated for PAH. This can be explained by the lower costs of drugs for PAH, and the short life expectancy (median survival of 2.8 years) of PAH patients if they remain untreated. Effective treatment options can thus result in a substantial number of life years gained, in contrast to the drugs for the slowly progressive, chronic metabolic diseases.

Remarkable differences are observed when comparing the ICURs from different economic evaluations in Fabry disease (€351 622 to €3 282 252 per QALY) [21, 25], Pompe disease (€153 405 to €1 043 868 per QALY) [19, 24], and Gaucher disease (€43 532 to €432 540 per QALY) [13, 14]. These differences can be attributed to the assumptions made; studies assuming survival with perfect quality of life [14, 21], or a huge increase in survival rate [19] upon therapy result in lower but less realistic ICURs.

## Discussion

We conducted a systematic review to identify cost-effectiveness and cost-utility studies for ultra-orphan drugs marketed in the EU and applied the CHEC-list to assess the quality of studies included. The challenges to perform adequate economic evaluations in the very small populations of ultra-orphan drugs are expected to be similar to the challenges in the broader group of orphan drugs and for drugs that are used for rare indications but have not received orphan drug designation. Only a limited number (*N* = 16) of studies on the cost-effectiveness or cost-utility of ultra-orphan drugs have been published, most of which were performed in the USA, the UK and the Netherlands. The large representation of studies from the UK and the Netherlands can be explained by the obligation to perform post-marketing economic evaluations in order to gain reimbursement. In the USA health technology assessments are not systematically conducted, but to an increasing extent orphans are part of the debate on pricing and cost-effectiveness [29].

Five of the 16 studies did not succeed to perform a real economic evaluation, or used an approach that was too simplistic to lead to a realistic estimate of the ICER/ICUR [14, 16, 21–23]. The latter either assumed the maximum possible benefit from therapy (immediate death in case of no treatment versus survival with perfect quality of life with treatment) [14, 21, 23], or based their estimate of effectiveness on only a very limited number of studies which probably led to an overestimation of treatment effect [16]. These studies highlight the fact that economic evaluations of ultra-orphan drugs are faced with many difficulties and limitations. The low number of patients and often slowly progressive, chronic character of the disease make a randomized clinical trial to estimate cost-effectiveness or cost-utility unfeasible. Pharmacoeconomic models using data from different sources such as literature, own patient cohorts and clinical experience are therefore more suitable [30]. Indeed, the majority of studies (*N* = 11) used models to estimate the cost-effectiveness or cost-utility ratio, including Markov-state-transition models in 7 studies, patient-level simulation models in 3 studies, and a decision tree in 1 study. A decision tree is the most simple model of disease progression. This type of model is primarily useful for short time horizon decisions where the likelihood of an event's occurrence is constant over time. It is in general not suitable for slowly progressive, chronic diseases although it has been used in the assessment of mercaptamin for the treatment of cystinosis [17]. The authors assumed that treatment would delay renal failure for 5 years. After renal failure all patients were assumed to follow the same disease course with dialysis and transplants, irrespective of previous treatment. Such a simplification of the disease course can be analysed with a decision tree, but the modelling choice for a chronic disease usually comes down to a choice between a cohort-level modelling approach such as a Markov-state-transition model, and a patient-level simulation model such as discrete event simulation (DES), which both allow for a more detailed representation of the disease course.

In patient-level simulation models, each patient is represented individually and the final outcomes are calculated by aggregating over all individuals [31]. This type of model has greater flexibility and allows for a better representation of heterogeneous patient cohorts than a Markov-state-transition model, since it allows for irregular time intervals between events. Also, the possibility to make the time to future events dependent on patient attributes including their history of previous events may result in a better reflection of the actual disease course. However, this type of model is in general more complex and needs more patient data to be able to reliably estimate specific effects and interactions of symptoms and complications on the progression of the disease. Kanters et al. used such a model to calculate costs and effects of alglucosidase alfa for the treatment of the infantile form of Pompe disease [24]. Only survival was modeled as event, making it a relatively simple model without the need for large patient numbers. Cost-effectiveness of bosentan has also been studied with patient-level simulation models [20, 27]. Again, only a limited number of events (clinical deterioration and/or death) were modelled. All 3 studies scored between 14 and 17 points. The question is whether a model with a very limited number of events accurately reflects the actual disease course. Complications that arise during the course of the disease were not taken into account while they probably result in a decrease in quality of life and an increase in costs. The model would become more complicated if these events were to be incorporated. The very small patient numbers in the case of ultra-orphan diseases may then limit the use of a patient-level simulation model, since it will be hard to gather sufficient information for reliable estimates of effects and interactions of symptoms and complications. Therefore, economic evaluations of ultra-orphan drugs will often use a cohort-simulation model, more specifically a Markov-state-transition model. This type of model has been used to estimate costs and effects of ERT for Gaucher, Fabry and Pompe disease [13, 19, 25], as well as drugs indicated for paroxysmal nocturnal haemoglobinuria [28] and PAH [15, 18, 26]. All studies scored between 14 and 19 points. In contrast to the patient-level simulation model, the Markov model represents the study population as a homogeneous cohort. It is particularly suitable to reflect the continuous risk of a disease over a long time period [30]. However, the suitability might be limited by the Markovian assumption of ‘no memory’, which means that the probability of transitioning from one state to the other entirely depends on the state the patient is in, regardless of the states the patient passed through the past.

In summary, the choice of which type of economic model to use is dependent on the type of disease that is described, the amount of data available, but also on the time and experience of the researcher. A Markov-state-transition model seems to be most suitable model for economic evaluations of ultra-orphan drugs considering the need for the incorporation of several disease states and the small patient groups. It should be noted here that we found a wide range of ICURs for the same drug even if the same type of model was used. This is at least partly caused by the many assumptions that had to be made due to limited data sets. To be able to build a model that comes as close as possible to the actual course of the disease under study, and to reduce as much as possible the uncertainty of the ICER/ICUR, it is of utmost importance to gather sufficient data. A crucial step in generating data is to establish disease-specific registries which include longitudinal data on all affected patients in the EU. Ideally, registries would start before drugs are marketed, to be able to generate data about the natural history of the disease. For this purpose, EU countries need to work in close collaboration. Existence of reliable data on the natural course and effectiveness of therapy enables researchers to conduct sound economic evaluations.

On the other hand, it has been argued that cost-effectiveness or cost-utility studies are inappropriate in the case of (ultra) orphan drugs since conventional criteria for cost-effectiveness will never be met. Indeed, considering studies using either a Markov-state-transition or patient-level simulation model, ICURs for different types of ERT ranged from €432 540 (Gaucher disease) to €3 282 252 (Fabry disease). Wyatt et al. calculated the number of QALYs to be generated each year in order for different types of ERT to be cost-effective which resulted in the impossible amount of 3.6-17.9 QALYs [22]. Consequently, most ultra-orphan drugs are not ‘cost-effective’ if standard health technology assessment procedures were to be applied to them, which was already stated in a previous paper by Drummond et al. [32]. It is highly unlikely that the ICUR could be decreased by the orders of magnitude required to make ultra-orphan drugs cost-efficient by current standards in the future. Although there is no clear consensus on what constitutes an acceptable cost-utility ratio, in the Netherlands a value of €80 000 per QALY for illnesses associated with a considerable burden has been suggested. However, so far, no drugs have been withdrawn from being reimbursed because of their unfavourable ICUR in the Netherlands. The National Institute for Health and Clinical Excellence (NICE) in England and Wales uses a threshold ICUR of £20 000 - £30 000 per QALY for non-orphan drugs [33]. The value of ultra-orphan drugs is assessed in a ‘Highly specialised technology (HST)’ programme, which is similar to the Single Technology Appraisal process [34]. The ICUR of £20 000 - £30 000 per QALY is not strictly adhered to when evaluating orphan drugs, and greater emphasis is applied to other factors such as ethics and lack of alternative treatments. In other countries in the EU (such as Ireland) there is no threshold ICUR value [35]. The lack of consensus on the threshold of the ICUR and absence of consequences for reimbursement in some countries may imply that cost-utility is not the only principle that has to be taken into account when assessing the value of orphan drugs. From a societal perspective, other (ethical) considerations may matter, such as the rarity and seriousness of the disease, the availability of alternative therapies and the cost to the patient if the drug would not be reimbursed [36]. These may be reasons to accept higher costs per QALY. Possibilities to deal with this are to apply variable cost-utility thresholds with a higher threshold for orphan drugs, or to value the health gain in a patient with a rare disease with a higher weight [5]. It is questionable, however, whether the QALY in itself is a suitable outcome measure in the case of ultra-orphan diseases. In most studies the EQ-5D (EuroQoL five dimensions) quality of life questionnaire is used to determine health status. Each observed health score profile on this questionnaire can be converted to a utility score ranging from - 0.594 (i.e., serious health problems with mobility, self-care, usual activities, pain/discomfort, and mood) to 1 (i.e., no problems at all). Patients with a slowly progressive, chronic disease will not be cured from one day to the other. In other words, the utility score will mostly not increase to perfect quality of life upon therapy. Instead, patients may feel less tired and better capable of working which is less well represented in the EQ-5D questionnaire. Consequently, the use of the QALY in its current form for the assessment of cost-utility of ultra-orphan drugs has been constantly discussed (see for example: http://www.rarediseaseblogs.net/author/cees-smit/). Others have argued that the QALY measure is even invalid [37]. Beresniak et al. asked more than 1300 subjects to express their preferences regarding combinations of different health states and time durations, which were subsequently compared to the results of the QALY formula (quality of life x number of life-years). They showed that observed and calculated utility values were significantly different. This might explain why costs/QALY estimates vary greatly. The authors argued that other approaches for health care decisions should be considered. Indeed, the costs/QALY approach has been rejected by the USA and Germany for ethical and methodological reasons [38, 39]. However, to remove an imperfect measure without replacing it with another might be not advisable [40].

Aside from using more suitable, representative ways to determine health status, bringing down costs would also lead to more acceptable ICERs/ICURs. The extremely high prices charged for new orphan drugs may cause an unsustainable pricing market that threatens the health care structure [41].

## Conclusions

Altogether, economic evaluations of ultra-orphan drugs are feasible if pharmacoeconomic modelling is used. The most suitable type of model seems to be a Markov-state-transition model. It should be realised, however, that most ultra-orphan drugs will not meet conventional criteria for cost-effectiveness. Still, ultra-orphan drugs are often reimbursed. Further discussion on the application of economic evaluations and their consequences in case of ultra-orphan drugs is therefore called for. The challenges described in this study may also apply to the broader group of (non-ultra) orphan drugs. However, this needs further study.

## Additional file

Additional file 1:
**CHEC-list scores of individual studies.** (PDF 28 kb)

## Abbreviations

CHEC: Consensus on Health Economic Criteria
DES: Discrete event simulation
EMA: European Medicines Agency
EU: European Union
EQ-5D: EuroQoL five dimensions
ERT: Enzyme replacement therapy
FOS: Fabry Outcome Survey
GBP: British pound
HST: Highly specialised technology
ICER: Incremental cost effectiveness ratio
LSD: Lysosomal storage disorders
NICE: National Institute for Health and Care Excellence
OMP: Orphan medicinal products
PAH: Pulmonary arterial hypertension
QALY: Quality-adjusted life year
UK: United Kingdom
USA: United States of America
