# Supplementary material for: The quality of economic evaluations of ultra-orphan drugs in Europe – a systematic review
Source: Orphanet J Rare Dis. 2015 Jul 30;10:92. doi: 10.1186/s13023-015-0305-y (PMC4520069; doi:10.1186/s13023-015-0305-y)
Supplement: Additional file 1: — CHEC-list scores of individual studies. (PDF 28 kb) [file 13023_2015_305_MOESM1_ESM.pdf]

CHEC

	Study														
	Rombach - Fabry	Connock - Fabry	Moore - Fabry	Dussen-Gaucher	Beutler - Gaucher	Kanters-Pompe	Castro - Pompe	Coyle - PNH	Connock - PNH	Soohoo - Cystinosis	Garin - PAH	Roman - PAH	Stevenson - PAH	Highland - PAH	Wlodarczyk - PAH
1. Is the study population clearly described?	(+)	(+)	(+)	(+)	(+)	(+)	(+)	(+)	(+)	(+)	(+)	(+)	(+)	(+)	(+)
2. Are competing alternatives clearly described?	(+)	(+)	(-)	(+)	(+)	(+)	(+)	(+)	(+)	(+)	(+)	(+)	(+)	(+)	(+)
3. Is a well-defined research question posed in answerable form?	(+)	(+)	(+)	(+)	(+)	(+)	(+)	(+)	(+)	(+)	(+)	(+)	(+)	(+)	(+)
4. Is the economic study design appropriate to the stated objective?	(+)	(-)	(-)	(+)	(-)	(+)	(+)	(+)	(-)	(+)	(+)	(+)	(+)	(+)	(+)
5. Is the chosen time horizon appropriate to include relevant costs and consequences?	(+)	(+)	(-)	(+)	(-)	(+)	(+)	(+)	(-)	(+)	(-)	(-)	(+)	(-)	(-)
6. Is the actual perspective chosen appropriate?	(+)	(-)	(-)	(+)	(-)	(+)	(-)	(-)	(-)	(+)	(-)	(-)	(-)	(-)	(-)
7. Are all important and relevant costs for each alternative identified?	(+)	(-)	(-)	(+)	(-)	(+)	(+)	(+)	(-)	(-)	(+)	(+)	(+)	(+)	(+)
8. Are all costs measured appropriately in physical units?	(+)	(+)	(-)	(+)	(-)	(+)	(+)	(+)	(-)	(+)	(+)	(+)	(+)	(+)	(+)
9. Are costs valued appropriately?	(+)	(+)	(-)	(+)	(-)	(+)	(+)	(+)	(-)	(+)	(+)	(+)	(+)	(+)	(+)
10. Are all important and relevant outcomes for each alternative identified?	(+)	(-)	(-)	(+)	(-)	(-)	(-)	(+)	(-)	(-)	(+)	(+)	(-)	(+)	(-)
11. Are all outcomes measured appropriately?	(+)	(-)	(-)	(+)	(-)	(+)	(+)	(+)	(+)	(+)	(+)	(+)	(+)	(+)	(+)
12. Are outcomes valued appropriately?	(+)	(+)	(+)	(+)	(-)	(+)	(+)	(+)	(+)	(-)	(+)	(+)	(+)	(+)	(-)
13. Is an incremental analysis of costs and outcomes of alternatives performed?	(+)	(+)	(-)	(+)	(+)	(+)	(+)	(+)	(+)	(+)	(+)	(+)	(+)	(+)	(+)
14. Are all future costs and outcomes discounted appropriately?	(+)	(+)	(-)	(+)	(+)	(+)	(+)	(+)	(+)	(-)	N/A	(+)	(+)	N/A	(+)
15. Are all important variables, whose values are uncertain, appropriately subjected to sensitivity analysis?	(+)	(+)	(+)	(+)	(-)	(+)	(+)	(+)	(-)	(+)	(+)	(+)	(+)	(+)	(+)
16. Do the conclusions follow from the data reported?	(+)	(+)	(+)	(+)	(+)	(+)	(+)	(+)	(+)	(+)	(+)	(+)	(+)	(+)	(+)
17. Does the study discuss the generalizability of the results to other settings and patient/client groups?	(-)	(+)	(+)	(+)	(-)	(-)	(+)	(-)	(-)	(+)	(+)	(-)	(-)	(+)	(+)
18. Does the article indicate that there is no potential conflict of interest of study researcher(s) and funder(s)?	(+)	(-)	(+)	(+)	(-)	(+)	(-)	(+)	(+)	(+)	(+)	(+)	(-)	(-)	(+)
19. Are ethical and distributional issues discussed appropriately?	(+)	(+)	(+)	(+)	(+)	(+)	(+)	(+)	(-)	(+)	N/A	N/A	N/A	N/A	N/A
Total CHEC-List (/19)*	18	13	8	19	7	17	16	17	9	15	15 (2)	15 (1)	14 (1)	14 (2)	14 (1)

* The number of items that were not applicable from the CHEC-List appears in parentheses. For example, discounting (Item 14) is not applicable for studies with a follow up of 1 year or less.

(+) yes/ study paid sufficient attention to aspect; (-) no/ item not reported; N/A, item not applicable.
